# Food waste tendencies: Behavioral response to cosmetic deterioration of food

**DOI:** 10.1371/journal.pone.0233287

**Published:** 2020-05-29

**Authors:** Vaneesha Dusoruth, Hikaru Hanawa Peterson

**Affiliations:** Department of Applied Economics, University of Minnesota-Twin Cities, St. Paul, Minnesota, United States of America; Washington State University, UNITED STATES

## Abstract

American households discard a significant amount of food that represent a sizable portion of their food expenditures. This study adds to our understanding of product attributes associated with food waste, with a focus on cosmetic deterioration during home storage. Specifically, we profile a sample of U.S. individuals by patterns of common food-related behaviors and determine the effects of product attributes on food waste tendencies at the point of consumption by distinct behavioral profiles. An interactive survey at the Minnesota State Fair (N = 333) was used to obtain measurements on food-related behavior and sociodemographic factors. The survey included a conjoint task to elicit food discard tendencies to construct the food waste proxy. The study considered cosmetic deterioration, date labels, implied shelf life, package size, and prices paid, in fresh, packaged spinach and ground beef products. Factor analysis and latent class modeling categorized the sample into two classes, revealing distinct food-related behavioral patterns. *Planners*, who constituted a slight majority in our sample, were likely to have established pre-shopping and in-store behavior and food management and cooking skills. *Extemporaneous Consumers* had inferior food handling routines and were less knowledgeable and skilled in the kitchen. Regression analysis using a random-effects tobit model showed *Extemporaneous Consumers* were prone to waste a greater portion of the spinach product than *Planners*. Otherwise, both classes showed similar increases in likelihood to discard the products, as their appearance deteriorated. Their tendency to waste increased with shorter remaining shelf life for spinach but not for ground beef, and was not affected by the date label type. Results suggest an intervention that targets a general audience designed to enhance people’s skills to discern edibility of food in home storage by manipulating sensory expectations from cosmetic deterioration could be impactful in efforts to curtail food waste.

## Introduction

Food waste has garnered much attention in local and global policy circles, as research continues to highlight its negative impacts on the environment. Evidence shows that wasted food places a huge burden on society in multiple ways, including opportunity costs of resources such as fresh water, cropland, and energy used to produce the food [1,2], and methane emissions generated from landfilled food aggravating climate change [1,3]. Currently, food scraps contribute to about 22% of the weight of material that goes to landfills in the United States [4], making food the single largest category of landfill waste. Decomposition of uneaten food alone accounts for 23% of all U.S. methane emissions [5].

Consumers disproportionately contribute to the food currently wasted and landfilled in industrialized nations [6,7]. A report by ReFED [6] estimates that in the U.S., household food waste accounts for 42% of 63 million tons of food wasted, followed by restaurants at 22%. Further, this makes up 51% of the total food waste that is landfilled. Buzby and Hyman [8] translate this food loss to 1% of household disposable income or equivalently consumer-level losses of food valued at $1.07 per day per household.

A growing literature shows that food waste results from multiple complex interactions and decisions that relate to food purchase and management, identifying a broad and varied set of sociodemographic, behavioral, and attitudinal factors that are both internal and external to individuals [9,10]. The array of studies highlighting multitude of similar but distinct factors has obscured understanding of universal drivers of food waste generation by individuals. Such fractured understanding leads to interventions that are inherently limited in scope—the scope falls short of what is needed to address this issue of global scale—and inhibits efforts to effectively prioritize interventions.

Appearance is a universal attribute to all products and establishes the first sensory impression of the item, majorly influencing its acceptability by confirming or disconfirming consumers’ sensory and hedonic expectations [11]. Industry practices have established consumer expectations for prototypical appearances of fresh produce [12,13,14], where consumers associate quality food with visual appeal [15,16]. Consumer have shown reduced acceptance of food items that deviate significantly from the prototypical shape [12], and promotional campaigns of “ugly” fruit and vegetable have called for acceptance of fresh produce with aesthetic deviations from the norm that have developed pre-harvest [17]. Damages and blemishes that occur post-harvest and appear at the point of purchase affect consumer perception of quality and reduce purchase intensions [18,19]. Products without any visual mars that have been purchased further undergo visual deterioration during home storage before they are actually consumed.

Our paper contributes to the literature in two ways. First, we profile U.S. subjects based on patterns of attitudes and behaviors related to household food-related routines such as shopping, planning, waste sorting, and other “home economics” skills. A handful of studies have aimed to categorize consumers based on food waste amounts or tendencies, or food-related practices [20,21,22]. We classify consumers by their general food-related behavioral patterns, including self-estimates of food they waste, and examine how food waste tendencies vary across groups. Second, we examine the role of cosmetic appearance of food on point-of-consumption decisions to discard, relative to other product attributes such as date labels that have recently been studied [23–26], by eliciting food waste tendencies using a conjoint task. Distinct from past studies that have focused on consumers’ acceptance of products with visual deviations from the norm that have occurred pre- purchase [12,19,27], we focus on the changes in food appearance during home storage while food remains edible. The use of a conjoint task to elicit food waste tendencies could minimize social desirability bias [28,29].

We designed an interactive consumer survey to collect observations on individuals’ attitudes, behaviors, and socio-demographic characteristics. Included was a conjoint task that simulated food handling scenarios at home to elicit food waste proxies. For the elicitation, we selected two products where appearance and expiration date are commonly factored into their consumption decisions, bagged spinach and ground beef. The survey was administered at the 2016 Minnesota State Fair, and 333 subjects participated. We applied principal component and latent analyses to group individuals by shared underlying traits, then used regression analysis to determine the relative roles of food-related attributes and socio-demographic characteristics on food waste tendencies and differences among groups.

The latent class model revealed that respondents fell into two, somewhat clichéd, classes: *Planners* and *Extemporaneous Consumers*. Making up 57% percent of the respondents, the *Planners* class consisted of people who were likely to have steady pre-shopping planning routines, diligent in-store behavior, consistent waste sorting practices, and good cooking and food management skills. In contrast, *Extemporaneous Consumers* of the second class were overall prone to have poor food planning and shopping routines and reported higher likelihood, for instance, to impulsively buy more food than needed in the store. Compared to *Planners*, they were less involved in activities such as recycling or composting and were generally less savvy and knowledgeable in the kitchen. Our regression results showed that *Extemporaneous Consumers* had higher tendencies to waste than *Planners* in the case of spinach, but two groups responded in a similar manner to product characteristics. That is, as cosmetic appearance of the food product deteriorated, people showed higher tendencies to consume less spinach and substantially less of ground beef, suggesting higher levels of potential waste, even though the product remained edible and safe to consume. Food waste tendency was higher for spinach as the remaining shelf life was shortened, but the effect was not statistically significant for ground beef. For both products, food waste tendency was unaffected by the type of date labeling or package size.

## Background and literature review

While food waste estimation is highly sensitive to methodologies employed [30], the literature finds that essentially about 31% to 40% of the food produced in the U.S. is wasted throughout the supply chain. In the U.S. and other developed countries, households currently generate the highest proportion of food discarded in the food supply chain. Consumers throw away over 25% of food and beverages they purchase [31]. Individuals do not appear to be fully aware of the environmental, economic and social consequences of the uneaten food they throw away [32].

Understanding contributors to food discarding habits has been a shared goal of consumer food waste research efforts, which were pioneered by Cox and Downing [33] in the UK and NRDC [15] in the U.S. A handful of reviews of recent developments in the literature reveal similar classification of factors [9,10,34,35], distinguishing behavioral factors in response to the food environment from factors that shape the environment. For example, do Carmo Stangherlin and de Barcellos [9] organize factors into personal (consisting of psychological and demographic factors), societal (originating from historical, regulatory, and supply chain sources), and behavioral (across various food-related routines such as shopping and cooking) categories. Schanes et al. [10] highlight the contribution of socio-psychological frameworks (such as the theory of planned behavior [36]) to the understanding of how personal factors impact food waste generated and of sociological frameworks (such as applications of the social practice theory [37]) in relating societal and behavioral factors to food waste amounts.

Because physical measurements of household food waste are costly to obtain, many of these studies prevalently have asked the subjects to self-report amounts of food discarded using a Likert scale [38,39] as proxies of household’s food waste behavior. Findings from a comparison between self-reported and measured food waste from curbside pickup support the use of self-reported measurements [40]; the relationship between behavioral determinants and both measurements were similar, although the degrees of relationship varied. In contrast, Elimelech et al. [41] found the objective and self-reported measurements led to opposite conclusions. Others created a proxy for food waste behavior from self-reported food waste amounts and food-related routines [42]. Wilson et al. [26] used a conjoint task to elicit simultaneously the proportion of food products individuals planned to consume and their willingness to pay for the product.

Studies have reported various demographic and psychological factors, such as motivations and barriers to reduce food waste, which are associated with household food waste tendencies. For instance, Neff et al. [43] note significant associations between food waste knowledge and demographics, where individuals aged 65 years or older reported greater knowledge of food waste, while households with children under 18 years of age expressed having less knowledge of food waste than other households. Yet, the findings of sociodemographic and psychological impacts on food waste generation seem context-specific, likely dependent on factors that are considered in respective studies. The most consistently reported sociodemographic effect is related to household size, where larger households generate more food waste at home, whether based on self-reported [44,45] or measured amounts [41,46], although per capita amounts could decline [15].

Meal planning and food shopping routines, as well as food handling skills, are found to be important predictors of food waste behaviors in most studies. For example, keeping a regular shopping schedule or checking inventories, as well as leftover reuse routines, are associated with smaller amounts of food waste [38]. Individuals with poor cooking and food management skills or lower food storage knowledge report higher levels of food waste [33,47]. The full series of food-related routines from pre-purchasing through eating and disposing affect food waste generated by households [48], with upstream routines found to be more influential than those at the downstream [44]. Similar to other personal factors, food-related routines are not always associated with food waste amounts, self-reported or measured [41,45].

A few studies have attempted to cluster or profile individuals by their food waste-related behavior and attitudes [20–22]. Romani et al. [20] and Gaiani et al. [21] segmented respective sample of Italian individuals. Using seven food-related behaviors generated from a factor analysis, Romani et al. [20] categorized individuals by self-reported amounts of food waste and waste intensions into “waster” “moderate” and “virtuous” clusters. They found that the clusters differed in terms of food-related behavior, personal/social norms, and moral attitudes. Gaiani et al. [21] used reasons why food is wasted to distinguish seven groups in their sample, describing them according to their sociodemographic characteristics and shopping, consumption, and waste behaviors. Pearson and Amarakoon [22] sorted Australian adults into four clusters according to the stages of change model based on self-reported efforts on food waste prevention and reduction. The clusters varied by sociodemographic characteristics with higher effort clusters having lower average body mass index.

Of industry practices affecting individual food behavior, package size, date labeling, and price are food product attributes that have conventionally been linked to food waste behavior. For example, packages that are too big or difficult to empty are identified as causes for food waste [49], and shorter shelf life implied by the food date leads to food wastage [26,49]. Studies have found distinct date label systems, such as “Use by” “Sell by” or “Best if Used by”, generate different amounts of potential waste [23,26], but Thomson et al. [25] concluded date labels had negligible effect on willingness to consume dairy products among Scottish individuals. Individuals, who pay attention to prices when shopping, have been reported as likely to waste less food than their counterparts [49].

Studies examining the role of food appearance in the context of food waste have largely focused on “ugly” or misshapen produce and its effect on purchase intensions. An exception, Jaeger et al. [50], examined the effect of the relative size of browning in apples on both point-of-purchase and point-of-consumption decisions. The findings suggest consumers have tolerance for modest deviations from prototypical appearance, where only significant deviations reduce purchase intensions [12,19,50]. Self-identity of being pro-environment may not matter, but awareness of food waste issues or boosting consumers’ self-perceptions may increase purchase intensions for abnormally shaped food [12,51]. Educating the public that “ugly” produce is safe to eat emerged as a critical recommendation from a series of focus groups [52]. Concerns for products’ shelf life increased the avoidance of browned bananas at point of purchase [53]. Since cosmetic deterioration directly affects consumers’ sensory expectations and perceptions [50,53], further studies are needed to understand how changes in visual appearance during home storage affects consumption decisions.

## Study design and methods

### Survey overview

The survey instrument was designed to collect information from individuals on relevant attitudinal and behavioral propensities based on the literature and to elicit food waste tendencies. The survey was administered in an interactive setting for a period of 5 days at the 2016 Minnesota State Fair, which was attended by nearly two million people during its twelve-day event. Volunteers recruited subjects for a total of about 30 minutes of their time, compensating them with a drawstring backpack with the University of Minnesota logo, a popular give-away item for human subject research projects conducted at the State Fair. Recruitment did not mention food waste to minimize self-selection bias in the study. The surveys were completed on electronic tablets. Our project staff worked closely with each respondent, explained the tasks at hand, and answered any clarifying questions. The study protocol was approved by the Institutional Review Board at the University of Minnesota.

### Elicitation of food waste proxy

To construct the food waste proxy for the study, the respondents were presented food products with varying attribute profile and asked to indicate the percentage of the presented food product they would likely consume as they are preparing meals, ranging from eating none to eating all. For each product profile, the question was posed as follows: *“Imagine you are in your kitchen to do some meal preparation using <food product>*. *The <food product> you took out is Product X*. *Thinking of all possible ways you are likely to eat <food product>*, *what percentage of this product are you and/or your household likely to eat*? *Answer*: *0%– 100% (sliding scale)*.*”* The food waste proxy score was computed by subtracting the responses in percent from 100, assuming that the smaller the proportion of products likely eaten, the higher the amounts potentially discarded.

We developed the food product profiles in consultation with food science experts to simulate realistic food handling decisions at home. In addition to cosmetic deterioration, our study focus, the following food product attributes were selected for the study from the attributes that have been associated with waste tendencies: expiration date type, days to expiration date, package size, and price purchased. We selected two products, packaged fresh spinach and ground beef, which are both sold with an expiration date and deteriorate visibly over time while their edibility is intact.

**Fig 1:**
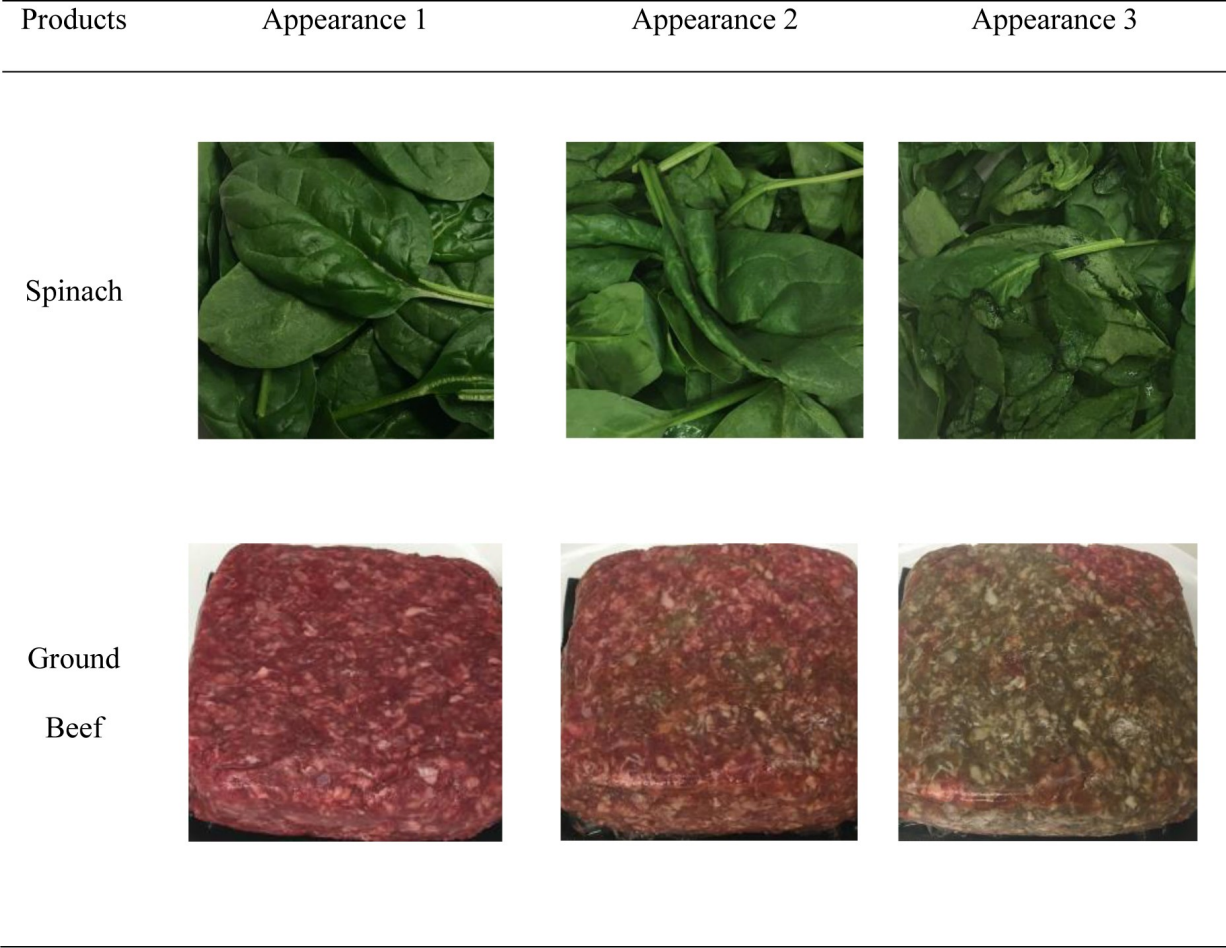
Cosmetic deterioration of spinach and ground beef.

Cosmetic deterioration was categorized into three distinct levels using photographic images. The *appearance level* of 1 suggests that the product was free from any cosmetic flaws. As the level progresses to level 3, there are multiple flaws in the form of browning in the case of beef and blemishes, spots, and wilting in the case of spinach. Respondents saw multiple images for each product from various angles. Deterioration was merely in appearance and products remained edible. Fig 1 displays the levels of cosmetic deterioration for the products, using one of the three visuals presented to respondents. Complete sets of visuals are available upon request.

Three types of expiration dates were considered: *Best by*, *Use by*, and *Best if used by*. These particular date types have been emphasized in the recent food waste literature and “*Best if used by*” is currently proposed as the preferred food date label [54]. Trading Partner Alliance, an industry group in the U.S. led by the Grocery Manufacturers Association and the Food Marketing Institute, has endorsed a two-tier system using the *Use by* terms for perishable products with potential safety implications or material degradation of critical performance, and *Best if used by* for all other packaged foods [55]. The other attributes were specified distinctly for spinach and ground beef. For days to expiration date, a *near*, *middle* and *far* expiration date for spinach implied 1, 3, and 7 days away respectively, and for ground beef, 1, 2, and 3 days away respectively. For package size, a large spinach product weighed 10 ounces and the small 5 ounces. The large ground beef weighed 2 pounds and small size was 1 pound. Price paid varied between $1.99 to $5.99 for spinach and between $4.79 and $15.99 for ground beef. These sizes and prices were selected to ensure familiarity with attributes at typical grocery outlets.

We used a fractional factorial method to develop twelve profiles for each product using orthogonal design. The product profiles were grouped into three blocks of four profiles, and each subject was presented with two randomly selected blocks of four profiles, one for each product, and both for spinach if they indicated that they did not eat meat. Thus, each subject evaluated eight product profiles, and their task was interrupted with a different set of questions between the blocks to minimize response fatigue.

### Factor analysis and latent model

The survey collected measurements related to shopping, purchasing, cooking and other food-related routines, which have been validated in the literature. The questions are included in the S1 Appendix. The factor analysis was applied to 35 measurements to reduce the dimension of factors. A minimum factor loading of 0.40 was considered in this analysis [56]. Factor loadings are the weights and correlations between each variable and the grouping pre-defined by researcher. The higher the load the more relevant the variable in defining the factor’s dimensionality [57]. Further, we evaluated the reliability and internal consistency of extracted factors through the Cronbach α, which is a lower-bound estimate of the reliability of the multiple measures of a construct [58]. This parameter can be viewed as the expected correlation among variables that measure the same concept. A score above 0.7 typically indicates high reliability [59,60].

These food-related behavioral factors were then used in a latent class analysis along with measurements of composting and recycling practices in the household (items 36 and 37 in the S1 Appendix). We presume that relationships among observed habits, behaviors and attitudes can be characterized into distinct consumer profiles which are unobservable to the researcher. A latent class analysis identifies such categorical latent variable through analyzing the structure of the statistical relationships among observed categorical variables [61]. The standard latent model computes the probability of the response patterns of the included measurements and uses a chi-square test to compare the sets of response patterns that were observed with the set of response patterns expected under the model. The Bayesian Information Criterion (BIC), derived by factoring in the log likelihood of the model, the number of estimated model parameters, and the total number of observations, is used to evaluate the latent model fit in terms of the number of classes [62,63]. The model with the lowest BIC is selected.

Individuals are assigned to classes based on the estimated conditional response probabilities based on the selected model, applying a modal classification rule, also defined as the highest posteriori probability rule [62]. These conditional response probabilities represent the probabilities that for each combination of latent class, observed variable, and response level for that item, that a randomly selected member of that class will make that response to that variable. The central idea is that class membership describes the composition of unobserved subgroups and captures different types of people based on their respective food-related behavioral patterns and attitudes.

### Regression analysis

We regress our proxy for food waste tendency on product attributes and sociodemographic factors for each product (bagged spinach and ground beef). The behavioral factors are captured by class membership. We examine whether food waste tendency varies across classes of individuals identified in the latent class. The data on our elicited measurements of food waste tendency and corresponding product attributes consists of multiple panel responses from participants. Thus, a regression model that accounts for panel-level effects is suitable, and we expect differences across participants to affect their food waste tendencies beyond individual characteristics we include in the model, which suggests including individual random effects [64].

We estimate the following regression model:

$$y_{its}=\beta_{0}+\sum_{s=1}^{S-1}s_{i}\beta_{s}+z_{t}\gamma_{0}+\sum_{s=1}^{S-1}s_{i}z_{t}\gamma_{s}+x_{is}\delta +u_{is}+\varepsilon_{its}$$ (1)

for *i* = 1, …, *n* individuals who are respectively members of class *s* = 0, …, *S*−1, where conjoint task *t* = 1, …, *n*_*i*_ and *s*_*i*_ is a binary indicator of class membership. The dependent variable *y*_*its*_ is the food waste tendency measured by the likelihood of product in conjoint task *t* being discarded by individual *i* in class *s*. The vector **z** represents a vector of product attributes which consists of the levels of cosmetic deterioration (appearance levels 1 through 3), expiration date type (best by, best if used by, use by), days to expiration, package size (large or small), and the price of product purchased. The vector **x** is a vector of demographic characteristics including age, gender, income, education level, race, household size, and presence of children in the household. **β**, **γ**, and **δ** are parameters. The coefficients on the interaction terms with the class indicator *s* will indicate whether food waste tendency varies across classes. The individual-level random effects, *u*_*is*_, are independently and identically distributed following a mean-zero normal distribution with a standard deviation of *σ*_*u*_, and the error term *ε*_*its*_ is independent and identically distributed following a mean-zero normal distribution with a standard deviation of *σ*_*e*_.

## Results

### Sample demographics

We begin by describing the overall demographic composition of the sample compared to the population of the state of Minnesota. Table 1 summarizes the characteristics of the 333 respondents. Most discrepancies are reasonable based on the study design. The subjects were recruited to participate in a research study at the University of Minnesota during their visits to the Minnesota State Fair at the end of August to early September in Saint Paul, Minnesota. The weather and outdoor nature of the Fair explain the smaller portion of individuals who are of age 65 and above. The subjects were considered eligible only if they were responsible for at least half of the food shopping for the household—which explains a higher proportion of female participants. In this case, female respondents make up about 66% of the sample compared to the Minnesota average of 50%. The income distribution is similar across the mid income levels, but underrepresents the lowest range and overrepresents the highest range. People with higher educational attainment than the state population participated. The sample consists of 9.0% of respondents with a high school degree or less while the state averages at 33%, while a larger proportion of the sample have a bachelor degree or higher in our sample. The sample reflects the overall racial diversity of Minnesota, although the representation of Black or African Americans is low. The household composition of participants is comparable to the state’s in terms of both the average number of household members and the percentage of households with children.

**Table 1:** Summary statistics of demographic variables.

		Survey participants (*N* = 333)	Minnesota population^a^
Age			
	18–24	12.6%	14.1%
	25–34	17.1%	19.1%
	35–44	17.4%	15.7%
	45–54	20.1%	17.7%
	55–64	21.3%	17.0%
	65 and over	11.4%	16.1%
Gender			
	Female	66.1%	50.3%
	Male	32.7%	49.7%
	Other	1.2%	
Household income			
	Less than $35,000	17.4%	27.6%
	$35,000 to $74,999	32.4%	32.0%
	$75,000 to $149,999	34.8%	29.5%
	$150,000 or more	15.3%	10.9%
Education			
	High school diploma or less	9.0%	33.4%
	Some college or associate’s degree	24.3%	34.4%
	Bachelor's degree or higher	66.6%	32.1%
Race			
	Asian	4.8%	4.5%
	Black or African American	3.0%	5.7%
	Other	6.3%	5.4%
	White	85.9%	84.3%
Household size (mean)		2.64	2.49
Children under 18 years of age present		24.0%	29.0%

^a^ Population data for the state of Minnesota from the American Community Survey 2016 5-year estimates. Age and education percentages are calculated for population 18 and over, not total population.

### Proxy measurement of food waste tendency

The scores expressed in percentage points (a scale of 0 to 100), calculated as 100 minus the elicited proportions of products that participants indicated as likely to consume, serve as proxy measurements of food waste tendency. Table 2 reports the summary statistics by product, for the entire sample and sub-samples identified by the subsequent latent class analysis. On average, participants responded to discard 30.7% of the presented bagged spinach products and 22.4% of the presented ground beef products. There is a wide variability among the responses, indicated by large standard deviations. About a third of responses for spinach (35.4%) indicated that they would not discard any of the products, compared to 52.5% of responses for ground beef. The mean scores among observations that were greater than 0% for the two products were nearly equal at 47%. Thus the difference in the calculated means of the food waste tendency scores between the two products can be attributed to the responses of 0%. Also, for both products, about 4.5% of responses indicated they would toss the entire product.

**Table 2:** Summary statistics of food waste tendency (score of 0 to 100).

		Mean	Std.Dev.	Number of observations	Observations equaling 0		Observations equaling 100	
					Number	(%)	Number	(%)
Bagged spinach								
	All	30.74	33.63	1,482	525	(35.4%)	65	(4.4%)
	*Planners*^a^	26.16	32.88	851	359	(42.2%)	41	(4.8%)
	*Ext*. *Consumers*^a^^,^^b^	36.91	33.65	631	166	(26.3%)	24	(3.8%)
Ground beef								
	All	22.35	32.80	1,178	618	(52.5%)	54	(4.6%)
	*Planners*^a^	19.15	31.12	659	367	(55.7%)	27	(4.1%)
	*Ext*. *Consumers*^a^^,^^b^	26.42	34.41	519	251	(48.4%)	27	(5.2%)

^a^ Classes identified by the subsequent latent class analysis.

^b^ Extemporaneous Consumers.

### Household food-related behaviors from factor analysis

The factor analysis applied to 35 items formed six variables gauging (i) pre-shopping routines, (ii) purchasing behaviors, (iii) reasons to discard food, (iv) self-reported amounts of food discarded, (v) cooking and food management skills, and (vi) tendencies to buy or prepare too much food to provide for the family. Table 3 displays the factor variables, the loadings on each construct, the related Cronbach α, and mean scores of individual items in parentheses. For instance, both the high factor loadings (>0.4) and the Cronbach α (0.72) for *Variable 1* suggest that the four measures of pre-shopping routines illustrate the essence of a person’s activities before grocery shopping. Making a list food purchases has a high loading of 0.80 implying that such a behavior is highly relevant in defining the pre-shopping routines factor’s dimensionality.

**Table 3:** Means, factor variables, loadings and Cronbach α (alpha)^a^.

VARIABLES		*Factor Loadings* ^b^
**Variable 1**		
*Pre-shopping routines*		
	I make a shopping list for our food purchases (3.85)	0.80
	I check our kitchen inventories before our shopping trips (4.03)	0.77
	I plan my meals in advance before a shopping trip (3.34)	0.74
	I have a regular schedule for my shopping trips (2.96)	0.65
	Cronbach α	0.72
**Variable 2**		
*Purchasing behaviors*		
	I tend to stick to my shopping list (3.52)	0.82
	I tend to buy products on the spot (3.21) ^c^	0.70
	I tend to buy food items in amounts according to my meal plans (3.58)	0.61
	Cronbach α	0.52
**Variable 3**		
*Reasons for throwing away food*		
*before meal prep*		
	The wrong food item was bought (4.13)	0.63
	It was passed best before/expiration date (2.75)	0.57
	The food looked ok but seemed no longer safe to eat (2.82)	0.55
	I bought too much or we already had item at home (3.40)	0.53
	The packaging was too large and contained more than I needed (3.11)	0.52
	The package was bad/broken (3.49)	0.47
	The food had visibly gone bad—rotten, sour, moldy, etc. (1.70)	0.46
*after meal prep*		
	The food did not turn out well (3.33)	0.63
	I did not want to save leftovers (3.84)	0.62
	I prepared too much (3.34)	0.58
	It was not possible to save leftovers (3.55)	0.56
	Saved leftovers had gone bad (2.06)	0.45
	Cronbach α	0.79
**Variable 4**		
*Amount of food discarded*		
*fresh foods (1 week)*		
	Fresh poultry/meats/seafood (1.71)	0.73
	Dairy and eggs (1.68)	0.66
	Fresh fruits and vegetables (2.46)	0.62
	Ready-to-eat deli (1.93)	0.57
	Fresh baked goods (1.97)	0.55
*shelf-stable foods (6 months)*		
	Frozen foods (1.67)	0.75
	Shelf-stable foods—pasta, cereal, etc. (1.50)	0.69
	Canned foods (1.32)	0.66
	Cronbach α	0.80
**Variable 5**		
*Cooking and food management skills*		
*cooking skills*		
	Preparing foods from raw/fresh ingredients (4.13)	0.70
	Cooking with leftovers/random ingredients to make a meal (3.79)	0.70
	Avoiding food getting burnt/ruined during cooking/preparation (4.06)	0.64
*food management skills*		
	Correctly resealing/repackaging opened products so they stay fresh (4.27)	0.75
	Knowing how to store different types of food products purchased (4.11)	0.75
	Eating foods that need to be eaten first (4.05)	0.67
	Cronbach α	0.79
**Variable 6**		
*Providing for the family*		
	I would prefer to buy more food than to run out (3.42)	0.88
	I would prefer to prepare more food than to run out (3.71)	0.88
	Cronbach α	0.70

^a^ Variable means reported in parentheses. The description corresponding to the Likert-scale can be found in the S1 Appendix.

^b^ Factor loadings are the weights and correlations between each variable and the factor.

^c^ Items were reverse coded.

While the Cronbach α of 0.52 suggests low reliability, the factor loadings for *Variable 2* imply that the measures are able to capture the factor’s dimensionality, particularly for sticking to the shopping list. When these items were combined with items in *Variable 1*, the results did not yield Cronbach α or factor loadings that met the minimum requirements, suggesting pre-shopping and in-store purchasing are distinct latent variables. The Cronbach α score of 0.79 shows that rationales for discarding food whether *before* or *after* meal (*Variable 3*) are internally consistent and reliable for the construct capturing activities and routines linked to food discarding. For example, amongst others, tendencies for buying the wrong item, not being able to save leftovers, or a package being broken leading to throwing away food are correlated and measure the same concept with good reliability. Self-reported amounts of food discarded are also closely related across all the different products (*Variable 4*), with an alpha of 0.80 indicating high reliability. Variable 5 captures a construct of food management and cooking skills which performs well according to the respective alpha and factor loadings. Finally, measures capturing agreement to preferring buying and preparing more food than running out are well correlated according to the Cronbach α score of 0.70.

### Latent class analysis

Using the six factors from the factor analysis in addition to two other variables that represent composting and recycling frequencies, the latent class analysis tested whether a group of unobserved classes (latent) validated the association among the included variables. Table 4 displays a summary of the Bayesian Information Criterion (BIC) for 1 to 4 classes.

**Table 4:** Latent class models and Bayesian Information Criterion.

Number of classes	Bayesian Information Criterion
1	1914.44
**2**	**1906.97**
3	2001.01
4	2095.28

The relative model fit evaluated by the BIC implies that the respondents fall into two classes (BIC = 1906.97). Fifty-seven percent of the sample make up, what we denote as, the *Planners* class. We call the other class, the *Extemporaneous Consumers*. Table 5 shows the conditional response probabilities for each response item (1) together with the related *p*-value (2). We compare these conditional probabilities in each case to assign a class membership for that specific response (3). Most probabilities are significant at the 5% level with the exception of a handful which are not used for interpretation for class membership.

**Table 5:** Conditional response probabilities to response items and class membership.

		(1)		(2)		(3)	
		Probability		P-Value		Class Membership	
		Pl.^a^	Ext.^b^	Pl.^a^	Ext.^b^	Pl.^a^	Ext.^b^
**Variable 1**							
*Pre-shopping routines*							
	Strongly disagree	0.01	0.09	0.23	0.00		×
	Somewhat disagree	0.04	0.24	0.04	0.00		×
	Neutral	0.13	0.29	0.00	0.00		×
	Somewhat agree	0.38	0.38	0.00	0.00	×	
	Strongly agree	0.45	0.00	0.00	0.73	×	
**Variable 2 **							
*Purchasing behaviors*							
	Strongly disagree	0.00	0.10	0.89	0.00		×
	Somewhat disagree	0.03	0.26	0.16	0.00		×
	Neutral	0.33	0.56	0.00	0.00		×
	Somewhat agree	0.43	0.08	0.00	0.03	×	
	Strongly agree	0.21	0.00	0.00	0.81	×	
**Variable 3 **							
*Reasons for throwing away food*							
	Very unlikely	0.02	0.03	0.08	0.06		
	Somewhat unlikely	0.18	0.24	0.00	0.00		×
	Neither likely or unlikely	0.39	0.54	0.00	0.00		×
	Somewhat likely	0.29	0.18	0.00	0.00	×	
	Very likely	0.12	0.01	0.00	0.39	×	
**Variable 4 **							
*Amount of food discarded*							
	Hardly any	0.71	0.36	0.00	0.00	×	
	Less than 10%	0.24	0.49	0.00	0.00		×
	More than 10% but less than 25%	0.03	0.12	0.04	0.00		×
	More than 25% but less than 50%	0.02	0.01	0.05	0.31		
	Over 50%	0.01	0.01	0.32	0.32		
**Variable 5 **							
*Cooking and food management skills*							
	Poor	0.00	0.01	0.96	0.16		
	Average	0.04	0.26	0.01	0.00		×
	Good	0.43	0.55	0.00	0.00		×
	Excellent	0.53	0.18	0.00	0.00	×	
**Variable 6 **							
*Providing for the family*							
	Strongly disagree	0.04	0.04	0.01	0.02		×
	Somewhat disagree	0.12	0.15	0.00	0.00		×
	Neutral	0.17	0.20	0.00	0.00		×
	Somewhat agree	0.44	0.38	0.00	0.00	×	
	Strongly agree	0.24	0.22	0.00	0.00	×	
**Variable 7 **							
*Composting habits*							
	About 1/2 of the time or less	0.71	0.89	0.00	0.00		×
	Most of the time/always	0.29	0.11	0.00	0.01	×	
**Variable 8 **							
*Recycling habits*							
	About 1/2 of the time or less	0.06	0.17	0.00	0.00		×
	Most of the time/always	0.94	0.83	0.00	0.00	×	

^a^ *Planners* Class

^b^ *Extemporaneous Consumers* Class

The results show that respondents who tend to either “Strongly Disagree,” “Disagree” or are “Neutral” about their pre-shopping routines such as making lists, and checking the inventories, largely fall into the *Extemporaneous Consumers* class. Those who tend to agree with these routines fall into the *Planners* class. Similar trends are noted for in-store or during shopping behaviors. Those who are in agreement with having fewer impulses in the store or sticking to their shopping lists fall into the *Planners* class.

When asked about reasons for food going to waste in the household, *Extemporaneous Consumers* report that they disagree with or are neutral about them. That is, on average, they do not throw away food because the package was bad/broken or too much food was prepared, for example. On the other hand, *Planners* tend to agree with throwing away food for various reasons such as being unable to save leftovers or buying the wrong item. The trend is reversed when it comes to self-reported amounts of food wasted, which include both perishables such as fruits, vegetables and meats as well as shelf-stable items such as frozen or canned goods. *Planners* report throwing away little amounts of food while *Extemporaneous Consumers* throw away an average amount.

*Planners* report that they prefer to buy or prepare more food than needed to provide for their families than to run out, while *Extemporaneous Consumers* mostly disagree with those statements. Regarding food management and cooking skills such as re-using leftovers or knowing how to store food items in the kitchen, *Extemporaneous Consumers* report either “Average” or “Good” skills. On the other hand, *Planners* report being “Excellent” at these home-economics skills. Finally, *Planners* report steady composting and recycling tendencies compared to their counterparts in the other class who do so less than half of the time.

### Regression analysis

The dependent variable of our analysis is the elicited measurement of food waste tendency, which is summarized in Table 2 by the two identified classes. For both products, average tendencies to discard for *Planners* are lower than those of *Extemporaneous Consumers*. A greater percentage of *Planners*’ responses indicate wasting no proportion of the food product compared to *Extemporaneous Consumers*, while a similar or greater percentage of response indicate tossing out the entire product.

Because of censored observations, we estimated a random-effects tobit model in Stata/IC 15.1, where lower and upper bounds were set at 0 and 100 respectively. Table 6 reports the regression results. The models for both products are statistically significant. The statistical significance of the between-subject variability (*σ*_*u*_) supports the specification of random effects. Table 7 reports average marginal effects and elasticities of product attributes and class difference on the expected value of the food waste tendency scores.

**Table 6:** Regression results: Tendency to waste products by product attributes^a^.

		Bagged spinach	Ground beef
Appearance			
	Level 2	5.174^**^	17.249^***^
		(2.617)	(5.249)
	Level 3	10.823^***^	52.410^***^
		(2.654)	(4.825)
Data type			
	Best by	0.256	5.961
		(2.713)	(4.790)
	Use by	3.819	3.540
		(2.784)	(5.307)
Days to expiration		-2.749^***^	-1.350
		(0.457)	(2.477)
Package size (large)		7.701	8.931
		(5.235)	(8.087)
Price paid		1.714	1.011
		(1.834)	(1.017)
Differences between classes			
	ClassEC	20.023^**^	13.218
		(9.720)	(16.825)
	ClassEC x Appearance_Level 2	-1.628	5.235
		(3.860)	(7.414)
	ClassEC x Appearance_Level 3	-0.727	6.289
		(3.898)	(7.005)
	ClassEC x Best by	-2.665	-0.971
		(4.001)	(7.168)
	ClassEC x Use by	-2.284	5.018
		(4.129)	(7.745)
	ClassEC x Days to expiration	0.375	-1.234
		(0.676)	(3.727)
	ClassEC x Package size	-1.725	-13.421
		(7.553)	(11.647)
	ClassEC x Price paid	-0.855	0.351
		(2.654)	(1.499)
Demographics			
	Age (Years)	-0.325^**^	-0.200
		(0.165)	(0.204)
	Gender (Male = 1)	9.679^*^	6.739
		(5.312)	(6.447)
	Household income ($10,000)	-0.233	-0.992^*^
		(0.468)	(0.574)
	Education	-8.959	-17.610^***^
	(Bachelor's degree or higher = 1)	(5.609)	(6.871)
	Race (Non-white = 1)	-7.241	15.269^*^
		(7.196)	(8.881)
	Household size	-2.503	4.082
		(2.264)	(2.699)
	Child presence	3.317	12.782
		(7.121)	(8.546)
Intercept		34.662^***^	-30.254^*^
		(12.179)	(16.976)
Sigma u (*σ*_*u*_)		41.927^***^	45.357^***^

		(2.121)	(2.968)
Sigma e (*σ*_*e*_)		24.325^***^	36.091^***^

		(0.677)	(1.401)
Log L		-4775.9	-3048.3
Wald Chi2 (H0: all coefficients = 0)		170.9^***^	277.19^***^
Number of observations		1,482	1,178
Number of individuals		333	295

^a^ Standard errors derived from asymptotic theory in parentheses.

*** p<0.01,

** p<0.05,

* p<0.1.

**Table 7:** Average marginal effects and elasticities^a^.

		Bagged spinach		Ground beef	
		Average marginal effect	Elasticity	Average marginal effect	Elasticity
Appearance					
	Level 2	3.083^**^		7.101^***^	
		(1.560)		(2.165)	
	Level 3	6.448^***^		21.576^***^	
		(1.583)		(2.015)	
Data type					
	Best by	0.153		2.454	
		(1.616)		(1.973)	
	Use by	2.275		1.457	
		(1.658)		3.677	
Days to		-1.638^***^	-0.217^***^	(0.556)	-0.06
expiration		(0.274)	-0.038	(1.020)	-0.11
		Bagged spinach		Ground beef	
		Average marginal effect	Elasticity	Average marginal effect	Elasticity
Package size (large)		4.588		3.677	
		(3.119)		(3.330)	
Price paid		1.021	0.129	0.416	0.209
		(1.093)	(0.138)	(0.419)	(0.211)
Extemporaneous		11.929^**^		5.442	
Consumer		(5.768)		(6.922)	
Demographics					
	Age	-0.194^**^		-0.082	
		(0.098)		(0.084)	
	Gender	5.767^*^		2.774	
		(3.148)		(2.650)	
	Household income	-0.139	-0.044	-0.408^*^	-0.211^*^
		(0.279)	(0.090)	(0.236)	(0.126)
	Education	-5.338		-7.250^**^	
		(3.333)		(2.811)	
	Race	-4.314		6.286^*^	
		(4.281)		(3.638)	
	Household size	-1.491		1.680	
		(1.350)		(1.107)	
	Child presence	1.976		5.262	
		(4.243)		(3.513)	

^a^ Standard errors based on the Delta method in parentheses.

*** p<0.01,

** p<0.05,

* p<0.1.

First, we turn our attention to the differences between classes. The class indicator variable (ClassEC) in the spinach results is statistically significant (p = 0.039), indicating that food waste tendency on average was higher among *Extemporaneous Consumers* than *Planners*, holding everything else constant. The coefficient on the class indicator is not statistically significant but positive in the ground beef results. The marginal effect, conditional on the food waste tendency score falling between 0 and 100, implies that given the same spinach product, *Extemporaneous Consumers* would on average discard 12 percentage point more of the product than *Planners* (Extemporaneous Consumer, Table 7). All interaction terms are statistically not different from zero, singly or jointly. Thus, *Extemporaneous Consumers* has a higher propensity to discard certain foods, but their response to product attributes that signal the edibility of food is no different from *Planners*. Thus, we examine the effects of products attributes on food waste tendency for the entire sample.

As cosmetic appearance deteriorates, we see a steady increase in the likelihood of food to be discarded. A slight but distinct decline in appearance from level 1 to level 2 increases the likelihood to discard by 3.1 percentage points for spinach (p = 0.048) and 7.1 percentage points for ground beef (p = 0.001), holding all other factors constant. A further decay to level 3 increases the likelihood to 6.4 percentage points for spinach (p < 0.001) and 21.6 percentage points for ground beef (p < 0.001), suggesting that one fifth of the ground beef product would be discarded for cosmetic decay even though it remains edible.

The only significant coefficient on date-related variables was the one on days to expiration in the spinach model. With each additional day closer to the labeled date, the likelihood of the spinach product to be discarded increases by 1.6 percentage point (p < 0.001), holding all else equal. Comparing the magnitude with that of appearance suggests that two additional days of shelf life have a comparable effect to offset the negative impact of the cosmetic decay from level 1 to 2. In terms of elasticities, a percentage decline in the number of days to expiration results in 0.22 percent increase in the food waste tendency, all else equal. Days to expiration had no discernable impact in the beef results. Thus, having more time until expiration is associated with reduced wastage propensities for produce but not for fresh meats. The date type did not affect the tendency to waste food for both products, which is distinct from the previous findings [23,24,26].

The results suggest higher wastage tendencies are associated with larger product sizes, but the results are not statistically significant (p = 0.141). This finding contrasts previous findings associating larger packaging with higher food wastage [26,49]. Similarly, the effects of prices paid on the amount of food discarded at the point of consumption were positive but not statistically significant (p = 0.350), unlike studies that have suggested consumers may find food purchased is too valuable to waste [49].

Sociodemographic variables were included in addition to individual random effects. The directions of impacts were largely consistent between the products, with the exception of race and household size. The tendency to waste was lower among younger respondents, all else equal, and the effect was statistically significant in the spinach results (p = 0.048). Male respondents had higher average tendency to waste by 5.8 percentage points than others in the spinach results (p = 0.067). Higher income earners had on average lower tendency to waste, which was significant in the ground beef results, suggesting a magnitude of 0.2% decline for a given 1% increase in household income (p = 0.093). Similarly, those with higher educational attainment had lower tendency to waste. The difference between those with a bachelor’s degree was 7.3 percentage points in the ground beef results (p = 0.010). Non-white respondents had on average 6.3 percentage points higher tendency to discard ground beef products (p = 0.084). Household composition had no impact on food waste tendency in either product.

### Limitations

The interactive survey tool was administered at the Minnesota State Fair implying that results from our consumer survey are subject to potential biases such as sample selection bias and measurement error [65,66]. To avoid sample selection biases, recruited respondents were told that the survey was concerned with food and food-related choices without mentioning food waste, which should minimize issues arising from topic-specific self-selection. But the study remains subject to the general bias of subjects with tendency to participate in a university-led research project. One key measurement error arises from the fact that the surveyed sample is not fully representative of the general population, which challenges the external validity of the results. The mixed results regarding the importance of socio-demographic characteristics in food waste behaviors [10] suggests a prospect that the different distribution of characteristics in the survey, while undesirable, would have minimal to moderate impact on the generalizability of the results.

Self-reported details about food and food-related behaviors such as shopping habits and amounts of food that potentially goes to waste in the household could be subject to information bias and more particularly recall bias [67,68]. These biases arise in part from respondents imperfectly remembering events and activities from the past. To reduce this type of bias, questions were carefully pre-tested and worded to ensure that respondents were able to recollect the intended events as accurately as possible [69]. Further, respondents were allowed plenty of time to reflect and think through the questions as they answered. Another concern is of respondents misreporting or misrepresenting measures such as amounts of food discarded in the household, which would be a form of measurement error. For instance, people may understate their amounts of food discarded if they place social value on that tendency. Our proxy for food waste behavior elicited using a conjoint task is probably less subject to social desirability bias than responses obtained from conventional survey items [28,29], and a future study can rigorously examine this.

## Discussion and conclusions

Our latent class analysis showed that individuals’ food and food-related routines can be typified into intuitive patterns. One type of behavioral patterns found in our sample were *Planners*, who are likely to have established pre-shopping food planning routines and steady in-store behavior. These individuals would keep track of their kitchen inventories, take a shopping list to the store, and, while in store, buy according to their list. *Planners* are more involved in environmental activities, such as recycling and composting, and report being knowledgeable and skilled in food management and cooking. Consequently, these *Planners* are aware of various reasons for why food gets discarded in their household, including buying or preparing too much food. Nevertheless, they report throwing out less food compared to their counterparts. In stark contrast, *Extemporaneous Consumers* are likely to have poor routines when it comes to meal planning and food shopping activities. They report being less knowledgeable and savvy when it comes to cooking and demonstrate lower food management skills in the kitchen. Compared to *Planners*, they tend to recycle or compost less frequently.

Our findings confirm other studies reporting a handful of profiles of food-related skills and behavioral patterns in our society [20,21]. Broadly speaking, some individuals are more comfortable with at-home, food-related skills than others. We could describe them as possessing a higher “food IQ” (intelligence quotient). Such food-related aptitude extends to food waste management, where food discarding decisions are made more deliberately resulting in lower amounts of food waste. As food-related skills and behavioral factors are all related, we do not need to be overly selective in describing or controlling for a myriad of known factors affecting food waste tendencies in prescribing policies. Moreover, we can expect for task- and situation-specific interventions such as those to promote self-efficacy in waste sorting at work [70] to have spillover effects across tasks and contexts. A similar effort to ours to synthesize behavioral factors applied to a more representative and larger sample of individuals in the U.S. and elsewhere would likely confirm patterns similar to *Planners* and *Extemporaneous Consumers*, while discovering additional or more nuanced patterns.

One salient finding from our study is that individuals with different behavioral profiles respond in a similar way to food product attributes. In particular, even if food products are fully edible, consumers are still likely to reject foods that had deteriorated in appearance. Moreover, these individual responses to specific attributes varies across products. The amount rejected for bagged spinach as it became visibly more wilted was modest, compared to the amount of rejection of ground beef as it displayed freezer burns and a few brown spots. Food waste tendency was much more responsive to remaining shelf life of the product indicated by date labels for spinach than for meat. For either product, date type, price, or package size had little impact, but this finding could be a result of the hypothetical nature of our study.

Previous studies suggest challenges with designing effective policy interventions to curtail household food waste, in part because practices that cause food waste are too fully integrated into daily routines to be changed with additional educational campaigns [34]. But there have been promising cases, such an educational intervention to enhance consumers’ perceived skills in planning meals [20]. Our findings call for a renewed attention to the prominence of visual cues in consumers’ sensory evaluation. Additional inquiry into how natural cosmetic deterioration of various perishable foods in home storage are associated with the sensory, hedonic, and risk expectations of individuals should provide insight into how effectively we could manipulate societal norms and strengthen the skills in how consumers assess the food’s acceptability at home.

Our findings suggest that people determine acceptability of food based on product attributes in largely the same way regardless of their food-related aptitude. However, it would be effective for policy interventions to be mindful of variation in the populace of their general food-related aptitude and proactively respond to segment-specific response in their implementation. For example, communications from campaigns need to be accessible to those who are generally less knowledgeable or familiar with at-home food handling.

## Supporting information

S1 Appendix (DOCX)
