# Supplementary material for: Food waste tendencies: Behavioral response to cosmetic deterioration of food
Source: PLoS One. 2020 May 29;15(5):e0233287. doi: 10.1371/journal.pone.0233287 (PMC7259764; doi:10.1371/journal.pone.0233287)
Supplement: S1 Appendix — (DOCX) [file pone.0233287.s001.docx]

**Appendix**

The measurements used in the factor analysis and latent class analysis were elicited from the following survey questions.

*Pre-shopping routines*

Thinking about your general food shopping trips, please answer the following questions: (1 = Strongly disagree, 2 = Somewhat disagree, 3 = Neither agree or disagree, 4 = Somewhat agree, 5 = Strongly agree)

1. I have a regular schedule for my shopping trips.
2. I plan my meals in advance before a shopping trip.
3. I make a shopping list for our food purchases.
4. I check our kitchen inventories before our shopping trips.

*Purchasing behaviors*

Thinking about when you are in the store, making your food purchases, please answer the following questions about how you shop: (1 = Strongly disagree, 2 = Somewhat disagree, 3 = Neither agree or disagree, 4 = Somewhat agree, 5 = Strongly agree)

1. I tend to stick to my shopping list.
2. I tend to buy products on the spot.
3. I tend to buy food items in amounts according to my meal plans.

*Reasons for throwing away food*

Thinking about the last month, what were the likely reasons for your household discarding food **before** meal preparation? (1 = Very unlikely, 2 = Somewhat unlikely, 3 = Neither likely or unlikely, 4 = Somewhat likely, 5 = Very likely)

1. I bought too much or we already had item at home.
2. The packaging was too large and contained more than I needed.
3. The wrong food item was bought.
4. It was passed best before/expiration date.
5. The package was bad/broken.
6. The food looked ok but seemed no longer safe to eat.
7. The food had visibly gone bad - rotten, sour, moldy, etc.

Thinking about the last month, what were the likely reasons for your household discarding food **after** meal preparation? (1 = Very unlikely, 2 = Somewhat unlikely, 3 = Neither likely or unlikely, 4 = Somewhat likely, 5 = Very likely)

1. I prepared too much.
2. The food did not turn out well.
3. It was not possible to save leftovers.
4. I did not want to save leftovers.
5. Saved leftovers had gone bad.

*Amount of food discarded*

In this section, we will ask you to estimate the amount of food before or after meal preparation that usually gets discarded in your household. **Please think of the edible portions of food (excluding parts such as peels, bones, seeds). Note that these food may be cooked food, leftovers, or unprepared foods.**

How much of the following you think is thrown away in your household of what you bought **in a typical week**? (0 = I do not buy these food products, 1 = Hardly any, 2 = Less than 10%, 3 = More than 10% but less than 25%, 4 = More than 25% but less than 50%, 5 = Over 50%)

1. Fresh fruits and vegetables
2. Fresh poultry/meats/seafood
3. Dairy and eggs
4. Fresh baked goods
5. Ready-to-eat deli foods

How much of the following you think is thrown away in your household of what you bought **during the last 6 months**? (0 = I do not buy these food products, 1 = Hardly any, 2 = Less than 10%, 3 = More than 10% but less than 25%, 4 = More than 25% but less than 50%, 5 = Over 50%)

1. Shelf-stable foods (pasta, cereal, etc.)
2. Frozen foods
3. Canned foods

*Cooking and food management skills*

Thinking about your regular cooking skills, how would you rate yourself on the following? (1 = Terrible, 2 = Poor, 3 = Average, 4 = Good, 5 = Excellent)

1. Preparing foods from raw/fresh ingredients
2. Cooking with leftovers/random ingredients to make a meal
3. Avoiding food getting burnt/ruined during cooking/preparation

Thinking about your regular food management skills, how would you rate yourself on the following? (1 = Terrible, 2 = Poor, 3 = Average, 4 = Good, 5 = Excellent)

1. Eating the foods that need to be eaten first
2. Knowing how to store different types of food products purchased
3. Correctly resealing/repackaging opened products so they stay fresh

*Providing for the family*

Please rate your stance on the following statements: (1 = Strongly disagree, 2 = Somewhat disagree, 3 = Neither agree or disagree, 4 = Somewhat agree, 5 = Strongly agree)

1. I would prefer to buy more food than to run out
2. I would prefer to prepare more food than to run out

*Composting and recycling habits*

Generally, does your household ...? (1 = Never, 2 = Sometimes, 3 = About half of the time, 4 = Most of the time, 5 = Always)

1. Compost
2. Recycle
